# Weakly Supervised Tumor Detection in PET Using Class Response for Treatment Outcome Prediction

**DOI:** 10.3390/jimaging8050130

**Published:** 2022-05-09

**Authors:** Amine Amyar, Romain Modzelewski, Pierre Vera, Vincent Morard, Su Ruan

**Affiliations:** 1 General Electric Healthcare, 78530 Buc, France; vincent.morard@ge.com; 2 LITIS-EA4108-Quantif, University of Rouen, 76800 Rouen, France; romain.modzelewski@chb.unicancer.fr (R.M.); pierre.vera@chb.unicancer.fr (P.V.); 3 Nuclear Medicine Department, Henri Becquerel Center, 76038 Rouen, France

**Keywords:** weakly supervised learning, class activation maps, tumor detection, radiomics, image classification, image segmentation

## Abstract

It is proven that radiomic characteristics extracted from the tumor region are predictive. The first step in radiomic analysis is the segmentation of the lesion. However, this task is time consuming and requires a highly trained physician. This process could be automated using computer-aided detection (CAD) tools. Current state-of-the-art methods are trained in a supervised learning setting, which requires a lot of data that are usually not available in the medical imaging field. The challenge is to train one model to segment different types of tumors with only a weak segmentation ground truth. In this work, we propose a prediction framework including a 3D tumor segmentation in positron emission tomography (PET) images, based on a weakly supervised deep learning method, and an outcome prediction based on a 3D-CNN classifier applied to the segmented tumor regions. The key step is to locate the tumor in 3D. We propose to (1) calculate two maximum intensity projection (MIP) images from 3D PET images in two directions, (2) classify the MIP images into different types of cancers, (3) generate the class activation maps through a multitask learning approach with a weak prior knowledge, and (4) segment the 3D tumor region from the two 2D activation maps with a proposed new loss function for the multitask. The proposed approach achieves state-of-the-art prediction results with a small data set and with a weak segmentation ground truth. Our model was tested and validated for treatment response and survival in lung and esophageal cancers on 195 patients, with an area under the receiver operating characteristic curve (AUC) of 67% and 59%, respectively, and a dice coefficient of 73% and 0.77% for tumor segmentation.

## 1. Introduction

To better appreciate the volume of interest in radiation oncology as well as the biological component of a tumor, radiomics has been proposed as a field of study that makes use of images [1]. Radiomic allows from an initial positron emission tomography exam (PET) the prediction of the survival of a patient and the response to radio-chemotherapy treatment, and therefore to help to personalize treatment [2,3]. The first step in a radiomic analysis is to localize tumor region for which radiomic features can be extracted. Manual segmentation is tedious and time consuming, especially in 3D. Deep learning is a very promising tool for automatic lesion detection in PET images, but due to their data-hungry nature, they require very large amounts of annotated images that are generally not available in the medical imaging field. Most of the segmentation methods use large annotated databases; however, annotating pixel-level tumor requires highly trained physicians and is time consuming. Moreover, physicians annotations can be subjective. In contrast, image-level labels indicating the presence of a lesion, or the type of cancer when they make the diagnosis are easy for the physicians and can be quickly obtained. Therefore, we propose an approach based on a weakly supervised learning (WSL), where image-level information is used to train a classifier based on a convolutional neural network (CNN) to predict the class label in a supervised learning way, and with a proposed appropriate transformation to detect and localize jointly tumors in an unsupervised way. Using only image-level labels to segment pixel-level image remains unexplored in PET images. To achieve this end, our strategy is to try to interpret how a neural network makes a classification decision.

The work on the interpretation of decision making by neural networks is an ongoing area of research. CNNs have yielded impressive results for a wide range of visual recognition tasks [4,5], especially in medical imaging [6,7,8]. Several approaches for understanding and visualizing CNN have been developed in the literature [9,10,11]. For instance, Zeiler et al. [10] used deconvolutional networks to visualize activation. The deep feature maps can be aggregated to extract class-aware visual evidence [12]. However, when fully connected layers are used for classification, the ability to locate objects in convolutional layers is lost. Various studies attempted to solve this problem using a fully convolutional neural networks (FCNs) such as Network in Network (NN) [13] and GoogLeNet [14]. Typically, conventional CNNs are first converted to FCNs to produce class response maps in a single forward pass. Although image-level class labels only indicate the existence of object classes, they can be used to extract indices for image segmentation, called class attention maps (CAMs) [12,15,16]. These class response maps can indicate discriminating regions in the image that allow a CNN to identify an image class. However, it cannot distinguish between the different objects in the image, which makes it difficult to segment accurately at the pixel level [17]. Different works have shown that although a CNN is trained to classify images, it can be used to locate objects at the same time [18,19]. Zhou et al. [11] demonstrated that CNNs can recognize objects while being trained for scene recognition, and that the same network can perform both image recognition and object localization in a single training. They showed that convolutional units of different CNNs layers can behave as object detectors despite the lack of object labels.

Ahn et al. [20] presented an approach for instance segmentation using only image-level class as label. They trained an image classifier model, and by identifying seed areas of object from attention maps, a pseudo instance segmentation labels were generated, then, propagated to discover the entire object areas with precise boundaries. Zhou et al. reported that local maximums in a class response map corresponds to strong visual cues residing inside each instance [21]. They created a novel architecture based on peak class response for instance segmentation using only the image-level label. First, a peak from a class response map is simulated; then, back-propagated and mapped to highly informative regions of each object instance, such as instance boundaries.

As for the outcome prediction, machine learning based methods are commonly used such as random forests (RFs) and support vector machines (SVMs) with or without a feature selection strategy [22,23,24]. The main disadvantage of these classical approaches is the need for an initial extraction of radiomic features using hand crafted methods, which usually yields a large number of features. In addition, handcrafted features are affected by many parameters [25] such as noise, reconstruction, and significantly by the contouring methods used. Recent studies aimed to develop classifiers based on CNNs which can automatically extract image features [26,27]. Our group has previously proposed a 3D CNN for esophageal cancer outcome prediction and has shown its effectiveness by comparing it to well known classical methods [28].

**Figure 1:**
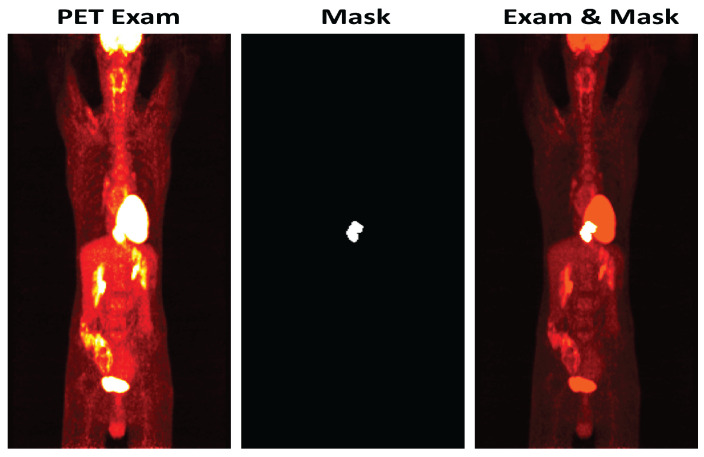
An example of a positron emission tomography (PET) image with esophageal cancer on the left in which the tumor is barely visible, and the same image on the right in which the location of the tumor is shown in a brighter color. It is not straightforward to learn the difference between tumor fixation and a normal fixation in a PET image.

Due to low PET image resolution, CAMs cannot be directly used as supervision for pixel segmentation since they cannot distinguish between physiological fluorodeoxyglucose uptakes (normal fixation/no tumor) and pathological uptakes (tumor), see Figure 1. The main concern of this method for processing PET images is the difficulty of identifying only the tumor region, because certain other regions of the image can also be identified as participating in the classification decision due to their strong visual information. In this work, we tackle the challenging problem of training CNNs with image-level labels for pixel-level tumor segmentation. We show that by using a CNN architecture with certain appropriate modifications and weak prior knowledge, we can transform the classification task into tumor localization in PET images. Specifically, we present a new method called PET-CAM for learning segmentation at the pixel level with class labels (at the image level) using the class response map, to make a CNN capable of segmenting the tumor at the pixel level but without pixel labels. Using only image-level labels to segment pixel-level image remains unexplored in PET images.

In addition, we propose to fully identify and locate tumor in 3D PET images using only two 2D MIP images for face and profile views, in order to reduce the complexity of the architecture and the learning time.

## 2. Materials and Methods

**Figure 2:**
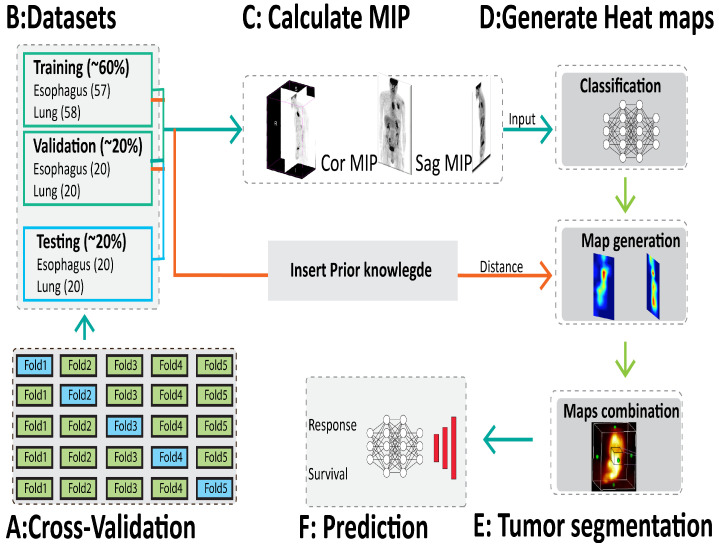
Study overview. (**A**) The dataset is divided five-fold for cross validation. (**B**) The data for each cross validation are divided into training, validation and testing. (**C**) Two MIPs in coronal and sagittal direction are calculated to be used as input for the neural network. (**D**) The neural network classifies the MIPs as lung or esophageal cancers and generates two heat maps corresponding to the two directions (coronal and sagittal). (**E**) The combination of the two heat maps allows for 3D tumor segmentation. (**F**) The segmented tumor is used as input to a second neural network for survival and response prediction.

Our method consists of two stages: segmentation of the tumor region and prediction of the treatment outcomes (Figure 2E,F). The core of our method is to develop a new method to generate heat maps with CAMs to locate the tumor region (Figure 2D). We propose a new loss function to improve the generation of CAMs, and therefore to locate the tumor more precisely. First, for each patient data we randomly define one point at the center of the tumor, which is considered to be prior knowledge. Then, we define a new loss function based on both the distance between the generated CAM at the current iteration and the central point, and the accuracy to classify the type of tumor. To that end, an eight-layer CNN is created to learn image-level labels and to generate an improved CAMs to locate correctly the lesions. After each feed forward of a mini batch of eight images, a probability of belonging to a class (tumor class) is generated and then a binary cross entropy loss function is calculated (Lclass). A CAM is generated for each image and a second loss for tumor localization is calculated. It is based on a distance between the CAM and the central point in the tumor (Ldistance). Finally, the back-propagation is performed in respect to both Lclass and Ldistance to update the weights.

### 2.1. Dataset

Our experiments were conducted on 195 PET images with lung (98) and esophageal (97) cancer. Patients underwent a whole body FDG PET/CT, at the initial stage of the pathology and before any treatment. The reconstructed exam voxel size was 4.06×4.06×2.0 mm^3^ and were spatially normalized by re-sampling all the dataset to an isotropic resolution of 2×2×2 mm^3^ using bicubic interpolation. The metabolic tumor volume (MTV) was segmented by a physician who manually defined a cuboid volume around the lesion and used a fixed threshold value of 40% of the maximum standard uptake value (SUVmax) in the cuboid. Tumor gray level intensities were normalized to have standard uptake volume (SUV) level between [0 30] and then translated between [0 1] to be used in CNN architecture. Data were split using a five-fold cross validation. We split the data into two groups to train and test the machine learning methods for each fold. One group was used for training the models (77 Oeso and 78 Lung) and one group for testing (40 patients). Furthermore, for the CNN, the training samples were split into a dataset of 2 groups, a train set (57 Oeso and 58 Lung) and a validation set (40 patients) (Figure 2B).

### 2.2. Maximum Intensity Projection

**Figure 3:**
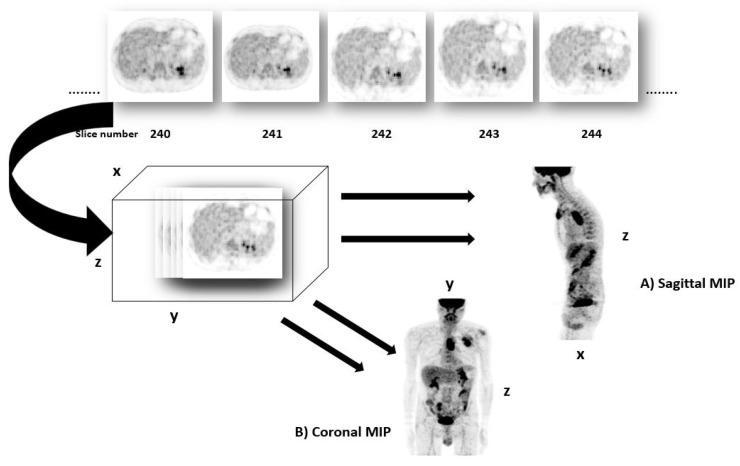
Maximum intensity projection (MIP) of a PET exam. (**A**) projection in Sagittal. (**B**) Projection in Coronal. The two MIPs in sagittal and coronal views allow providing a better context than one MIP view and to maintain a link to the x, y and z coordinates.

Maximum intensity projection (MIP) is a 2D image that represents 3D image for fast interpretation in clinical applications [29]. Our idea is to use MIP to deal with 3D images, allowing on the one hand to greatly reduce the complexity of the networks and avoids over-fitting due to the small size of the medical image data set, and on the other hand to keep useful 3D information. Two MIPs calculated from opposite points of view are symmetrical images if they are rendered by orthographic projection. MIP imaging is used routinely by physicians in interpreting PET images. It can be used for the detection of lung nodules in lung cancer screening programs, for example. MIP enhances the 3D nature of these nodules, making them stand out from pulmonary bronchi and vasculature [30]. This technique is computationally fast; thus it can be used for image classification, to classify the different pathologies such as lung cancer or esophageal cancer. However, the radiomics features obtained from MIP images are not rich enough to predict the outcome of treatment and survival, due to the loss of depth information. To obtain a 3D tumor region, we propose to use sagittal and coronal MIPs to have different views, as shown in Figure 3. The strategy is to use 2D images to obtain 3D tumor region, allowing speeding up the tumor localization, since a 3D activation map generation is time consuming and is hard to train with limited resources.

### 2.3. New Design of Class Activation Map (CAM)

**Figure 4:**
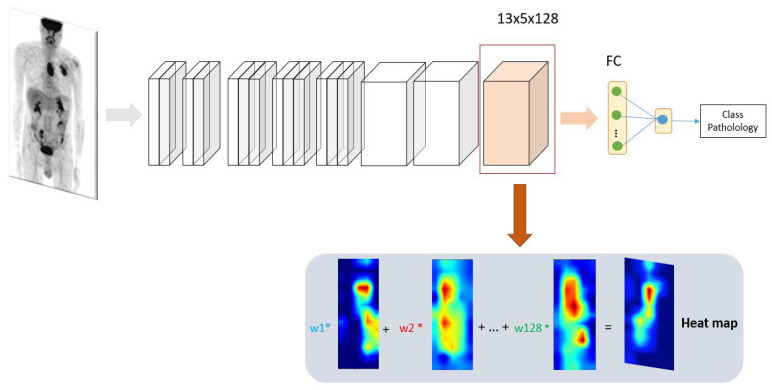
Heatmap generation using convolutional neural network. The MIP is used to predict the pathology. A heat map is generated from the last convolutional layer. The heat map allows to highlight class-specific regions of the image.

Interpreting machine learning models is a key element towards making it easier for physicians to embrace these methods. To interpret a convolutional neural network, we can produce a heat maps to identify locations of zones in images that contribute the most to the network classification decision. In this work, we aim to generate heat maps using CAM which is one technique for producing heat map to highlight class-specific region of images [15]. It is a key step in our method, since it will be used to recover the entire tumor area in a PET image. When a MIP image is passed to a convolutional neural network, it is passed through a series of layers. The early layers in a CNN capture low-level features while the later layers capture higher level visual information that is relevant to the classification task. Finally, we flatten the last convolutional layer, and then passed to a fully connected layers provide a certain probability of belonging to the esophageal class or lung one, see Figure 4.

In a CNN-based classifier, once the features are flattened, the spatial information is lost. Therefore, if we want to visualize the features that the model has picked up from the image, we must visualize the features before the flattening occurs. We thus take the feature maps’ last convolutional layer to generate the heat map. These feature maps are much smaller in size than the input. Typically, the width and high of CAMs are 1/33 of that of the input image and the number of feature maps are the same as the output of the last layer (128). We note the total number of feature maps in the last layer by *D*. To go from these feature maps with size of 13 × 5 to a heat maps over the whole image, we need to unpack these feature maps. Let $f^{i}$ be the *i*th feature map. For each feature map $f^{i}$, a weight *w* is associated to it, where *i* = 1, …, *D*. Then, a pre-heat maps is obtained by adding each feature map multiplied by its weight as in (1):

(1) $$pre\_hmap=\sum_{i=1}^{D}\left[w^{i}f^{i}\right]$$

Each feature maps contains 13×5 elements (65 in total), where ${f^{i}}_{j,z}$ is (*j*,*z*) element of the *i*th feature map, where *j* = 1, …, 13 and *z* = 1, …, 5. To obtain the wights *w* for each of these feature maps, we calculate the influence of ${f^{i}}_{j,z}$ on the output $\hat{y}$, by computing the partial derivative of $\hat{y}$ with respect to each feature in $f_{i}$, such as:

(2) $$I=\frac{\partial \hat{y}}{\partial {f^{i}}_{j,z}}$$

Then, $w^{i}$ is calculated by taking the average of the feature influences at each j,z position as in (3):

(3) $$w^{i}=\frac{1}{N}\sum_{j=1}^{J}\sum_{z=1}^{Z}\frac{\partial \hat{y}}{\partial {f^{i}}_{j,z}}$$

where *N* is the number of elements in the feature map, *J* is the width and *Z* is the height. Finally, we keep only features with positive influence. Thus, we apply a *ReLU* function to keep only positive values. The heat map is finally obtained by:

(4) $$h\_map=ReLU(\sum_{i=1}^{D}\left[w^{i}f^{i}\right])$$

where ReLU(X) is defined as:

(5) Relu=max(0,X)

Because the heat map is generated at a low resolution of 13×5, we interpolate it to adapt it to the size of the MIP images. In our application, the heat map can be generated for 2 different types of cancer, corresponding to two classes: lung cancer and esophageal cancer. Let *C* denote the class ∈ {lung, esophagus}. Therefore, from (3) and (4) we have:

(6) $${w^{i}}_{C}=\frac{1}{N}\sum_{j=1}^{J}\sum_{z=1}^{Z}\frac{\partial {\hat{y}}^{C}}{\partial {f^{i}}_{j,z}}$$

(7) $$h\_map^{C}=ReLU\left(\sum_{i=1}^{D}\left[{w_{C}}^{i}f^{i}\right]\right)$$

The obtained heat maps will be used afterwards to calculate a new loss function based on distance between the inter pixels within the heat maps and the tumor region (see next section). Then, a new heat maps will be created through the backpropagation with two losses: classification loss and distance loss.

**Figure 5:**
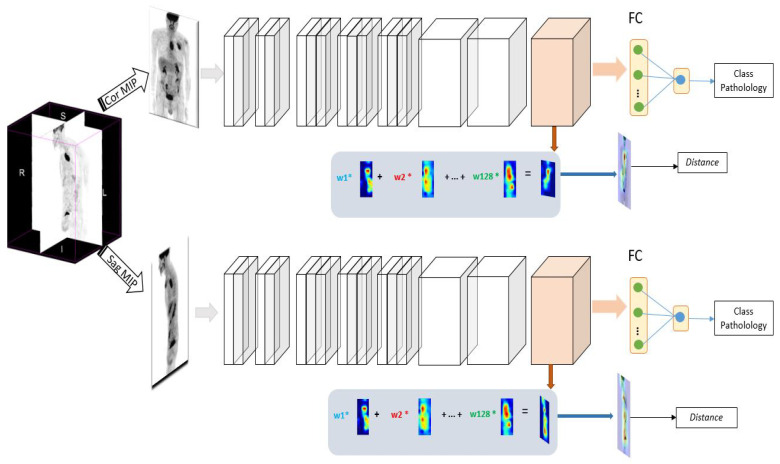
Our proposed architecture. The neural network learns to classify the type of cancer from two 2D MIP images (sagittal and coronal). The generated heatmap is back-propagated and corrected to identify accurately tumor regions.

We introduce this novel loss function to prevent heat maps from further resolution drop. A large loss indicates that the current representation of the networks does not accurately capture the lesion’s visual patterns, and it is, therefore, necessary to provide an additional mechanism for self-improvement through back-propagation. The resulting architecture (see Figure 5) is a novel convolutional neural network with an attention feedback, having an improved localization capability.

#### 2.3.1. Classification

The resulting set of feature maps, encloses the entire spatial local information, as well as the hierarchical representation of the input. Then, each feature map is flattened out, and all the elements are collected into a single vector *V* of dimension *K*, providing the input for a fully connected hidden layer, called *h*, consisting of *H* units. The activation of the *i*(th) unit of the *h* hidden layer is given by:

(8) $$h_{i}=g\left(b_{i}+Wh_{i}*V\right)\;\mathrm{with}\;i=1,\ldots ,H.$$

The multilayer perceptron consists of a two Dense layers with 128 and 64 neurons, respectively, with a dropout of 0.5 and the activation function elu. The last layer is a Dense layer with one neuron for image classification using a sigmoid activation. The binary cross entropy is used as the loss function (*Lclass*):

(9) $$Lclass=-\frac{1}{n}\sum_{i=1}^{n}\left[y_{i}\log\left({\hat{y}}_{i}\right)+\left(1-y_{i}\right)\log\left(1-{\hat{y}}_{i}\right)\right]$$

where *n* is the number of patients, *y* is the cancer lung label (binary, 1 if the patient has lung cancer, 0 if it is esophageal cancer) and ${\hat{y}}_{ij}$∈ (0,1): ∑_*j*_${\hat{y}}_{ij}$ = 1 ∀ *i*,*j* is the prediction of a lung cancer presence.

#### 2.3.2. Distance Constraint Using Prior Knowledge

Unlike the visual explanation Grad-CAM proposed by Selvaraju et al. [15], we introduce a second loss based on a distance between the central point *p* defined within the tumor region and the points in the generated heat maps, defined as follow:

(10) $$Ldistance=\sqrt{\sum_{i=1}^{m}\left|q_{i}-p\right|}$$

where *q*_i_ notes a point *i* and *m* is the number of points in a heatmap. This second loss function makes it possible to correct the errors of the heat maps generated through the distance constraint. In fact, instead of focusing on the discriminating regions, which may include information other than the location of the tumor for classification, the heat map is regularized with the distance constraint to emphasize the region of the tumor and at the same time keep a good classification.

The global loss function (*loss glob*) for the 2 tasks is defined by:

(11) loss_glob=Lclass+αLdistance

where α is a constant weight coefficient. We used an α = 1 in our study.

### 2.4. Segmentation

**Figure 6:**
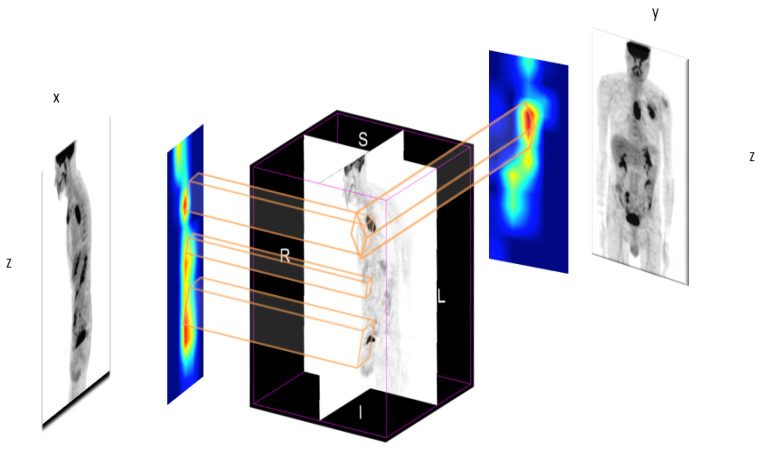
Segmentation: the 3D tumor region from the two 2D heat maps. Coronal heat map allows to retrieve y and z axis, while sagittal heat map return x and z axis. The tumor is selected by the intersection of the two heat maps.

Once we obtain the heat maps for sagittal and coronal MIP views, we retrieve the lesions mask on the 3D image. Sagittal MIP allows to retrieve y and z axis, and coronal MIP the x and z axis. Combining the 3 coordinates finally results in the 3D volume of the tumor, see Figure 6.

### 2.5. Prediction

**Figure 7:**
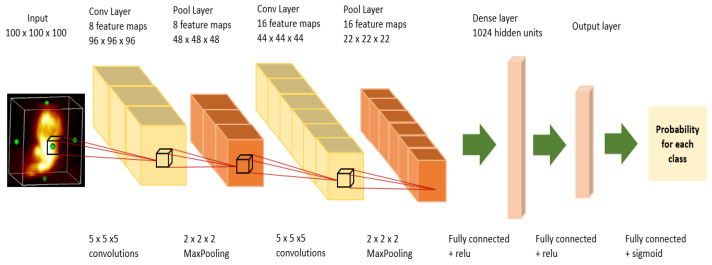
3D RPET-NET architecture composed by two 3D convolutional layers followed by 3D pooling layers and two dense layers.

Once we obtain the 3D tumor region, we conduct a radiomic analysis to predict patient survival and treatment outcome. We use 3d-rpet-net [28], a CNN classifier based on two 3D convolutional layers and two fully connected layers to conduct radiomic analysis (see Figure 7). The same model is applied on the 3D volumes segmented by a physician and those obtained by our automatic method in order to compare their performance.

## 3. Experiments

### 3.1. Setup

**Figure 8:**
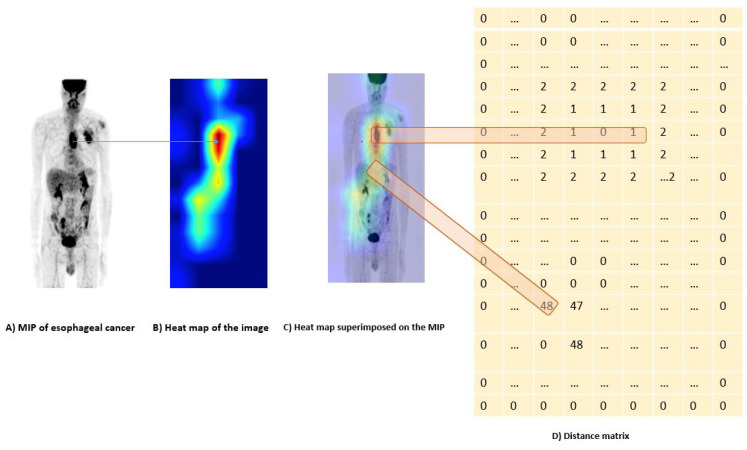
Distance matrix between *p* at the center of the tumor and the points *q*${}_{i}$ generated by the heat map. (**A**) is a Coronal MIP for a patient with esophageal cancer. A point *p* is randomly defined at the tumor region. (**B**) is the heat map generated using our proposed model. (**C**) shows the overelay of the MIP and the heat map. (**D**) is the distance matrix showing the distance between the points *q*${}_{i}$ generated by the heat map and the point *p*.

We firstly generated the MIPs for the front view and for side view. MIP is a 2D image that summarizes 3D images for fast interpretation. Tumor gray level intensities were normalized to have SUV level between [0 30] and then translated between [0 1] to be used in CNN architecture. The neural network is trained to classify the type of cancer: esophageal vs. lung cancers. For each mini batch, CAMs are generated, backpropagated and corrected via a distance function (see Figure 8), to differentiate tumor regions from normal regions. Then, the two resulted corrected CAMs, for face and profile view are combined to retrieve the 3D tumor.

Two experiments are conducted to evaluate our model.

Experiment 1: The first experiment consisted of segmenting the lesions on the 3D PET images for patients with esophageal cancer and lung cancer, using only 2D MIPs. The results were compared to the state-of-the-art method U-NET [31], which is commonly used in medical imaging for fully supervised segmentation, and CAMs without prior knowledge.

Experiment 2: The second experiment consisted of radiomics analysis. We predict the response to treatment for esophageal cancer, and the patient’s survival for lung cancer. The response to treatment was evaluated three months after the end of treatment, and the overall survival (OS) used for the prognostic study was estimated at three years after the end of the treatment.

### 3.2. Implementation

The model was implemented using python with pytroch deep learning library, and trained for 2 days on nvidia p6000 quadro GPU with 24 gb.

## 4. Evaluation Methodology

We divide the dataset into three groups: training, validation, and test. For a fair comparison, all the methods were trained, validated and tested with the same group of data. The performance of the models were evaluated using the dice coefficient for the segmentation task, and the accuracy (Acc), sensitivity (Sens), specificity (Spec) and area under the ROC curve (AUC) for the classification, such as:

(12) $$\mathrm{Sens}=\frac{\mathrm{TP}}{\mathrm{TP}+\mathrm{FN}}$$

where TP is the true positives, FN is the false negatives and TP + FN is the number of patients classified positively.

(13) $$\mathrm{Spec}=\frac{\mathrm{TN}}{\mathrm{TN}+\mathrm{FP}}$$

where TN is the true negatives, FP is the false positives and TN + FP is the number of patients classified negatively.

(14) $$\mathrm{ACC}=\frac{\mathrm{TP}+\mathrm{TN}}{\mathrm{TP}+\mathrm{FN}+\mathrm{TN}+\mathrm{FP}}$$

## 5. Results

Table 1 summarizes the mean and standard deviation values across five cross validation for tumor segmentation for both esophageal and lung cancers. Five methods were compared to our proposed model with: U-NET, SegNet and ResUnet using fully supervised learning, CNN and FCN with CAMs without prior knowledge.

Table 2 shows results of radiomic analysis for the prediction of patient’s treatment three months after the end of radiochemotherapy for esophageal cancer, and the prediction of three years’ survival for patients with lung cancer.

All the methods were compared based on the ability to detect accurately the tumor and to conduct a radiomic analysis. The performances are measured by accuracy, sensitivity, specificity, and the area under the ROC curve. The results were obtained using a five-fold cross validation. The best results for segmentation were obtained using our proposed model for both lung (dice = 0.77 ± 0.07) and esophageal (dice = 0.73 ± 0.09) cancers.

**Figure 9:**
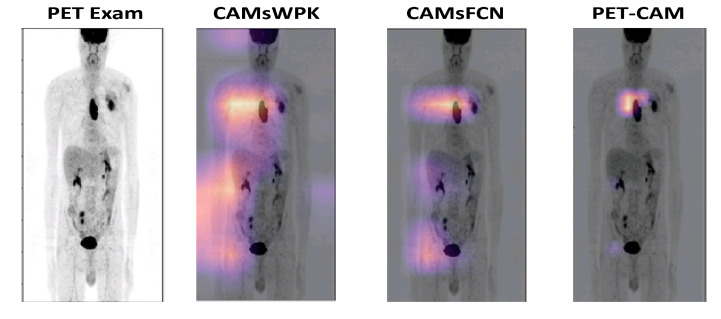
Comparison between different models. From left to right: PET exam, CAMs without prior knowledge using CNN, CAMs without prior knowledge using FCN, our proposed PET-CAM model. PET-CAM tends to focus on a region near the region of interest, while standard CAM may look at different organs or outside the body.

For radiomics, 3d-rpet-Net with manual segmentation was not statistically significantly different from our model for esophageal cancer (*p* = 0.59) and for lung cancer (*p* = 0.63). Our model tended to have a better sensitivity for esophageal and a better specificity for lung cancer with no significant differences. Figure 9 shows a CAM of one patient. PET-CAM tends to focus on the region of interest while CAM without prior knowledge (constraint) focuses also on other regions.

## 6. Discussion

In this study, a new weakly supervised learning model was developed to localize lung and esophageal tumors in PET images. It uses two fundamental components: a new class activation map to locate the tumor and a new loss function to improve localization precision. The model could detect tumors with better accuracy compared to fully supervised models such as U-NET, or classical CAMs. Our model outperformed other methods in terms of the dice index. As for radiomic analysis, 3d-rpet-net with manual segmentation has shown slightly better results compared to our WSL model in radiomic analysis. However, it is based on manual pixel-level annotations of tumor, which requires an expert physician and also is time consuming.

The first step in radiomic analysis is the segmentation of the lesion. This process could be automated using computer-aided detection (CAD) tools. State of the art U-NET have shown very good performance for image segmentation in different fields. In medical imaging, it is usually used as a backbone for tumor or organs segmentation. The main drawback of U-NET is the need for a large dataset to work efficiently. Fully labeled dataset is very hard to obtain in the medical imaging field due to several reasons such as: protection of the patient’s privacy, establishment of a specific protocol for data recovery, the need for an expert physician for data labeling, etc. Moreover, physician’s labels are subjective and prone to error.

By detecting the tumor with 2D MIP images for face and profile views, we can obtain x, y, and z coordinates to segment the 3D image. The segmentation in the 3D images were used to conduct a radiomic analysis with state-of-the-art results. This simple and yet powerful technique can be integrated in future workflow/software dedicated to automatic analysis of PET exams to conduct radiomic analysis.

One of the major challenges in deep learning is models’ generalizability. Often, radiomic studies are single-center studies on data from the same center and the same machine. Another major challenge of radiomics is the obtaining of a robust predictive and prognostic signature that works on data from different centers. One of the limitations of our study is the use of single-center data. In the future, we will test our model on larger data from different hospitals to improve its robustness.

## 7. Conclusions

PET-CAM model enables 3D lung and esophageal lesions segmentation from only two 2D MIP images. The resulted segmentation could be used in a radiomic analysis to predict treatment outcome for esophageal cancer and survival for lung cancer. We showed that by training a neural network with weakly annotated data it allows to achieve state-of-the-art results in both tumor segmentation and outcome prediction. 

## Figures and Tables

**Table 1:** Results for 3D segmentation. WPk: without prior knowledge. CAM: class activation map.

	Method	Dice	IOU
Esophageal cancer	U-NET [31]	0.42 ± 0.16	0.32 ± 0.03
	SegNet [32]	0.57 ± 0.14	0.45 ± 0.05
	ResUnet [33]	0.55 ± 0.19	0.45 ± 0.03
	CAMsWPK	0.53 ± 0.17	0.42 ± 0.04
	CAMs & FCNs	0.73 ± 0.12	0.63 ± 0.05
	PET-CAM	0.73 ± 0.09	0.62 ± 0.03
Lung cancer	U-NET [31]	0.57 ± 0.19	0.45 ± 0.04
	SegNet [32]	0.69 ± 0.12	0.59 ± 0.04
	ResUnet [33]	0.66 ± 0.14	0.55 ± 0.03
	CAMsWPK	0.63 ± 0.14	0.51 ± 0.05
	CAMs & FCNs	0.73 ± 0.12	0.63 ± 0.04
	PET-CAM	0.77 ± 0.07	0.65 ± 0.03

**Table 2:** Results for radiomics analysis. WPk: without prior knowledge. Ms: manual segmentation.

	Method	Accuracy	Sensitivity	Specificity	AUC
Esophageal cancer	U-NET [31]	0.47 ± 0.07	0.67 ± 0.22	0.31 ± 0.21	0.48 ± 0.24
	SegNet [32]	0.57 ± 0.05	0.69 ± 0.19	0.44 ± 0.22	0.55 ± 0.10
	ResUnet [33]	0.53 ± 0.08	0.57 ± 0.23	0.47 ± 0.23	0.55 ± 0.17
	CAMsWPK	0.57 ± 0.03	0.61 ± 0.28	0.56 ± 0.24	0.53 ± 0.26
	MS	0.72 ± 0.08	0.79 ± 0.17	0.62 ± 0.21	0.70 ± 0.04
	CAMs & FCNs	0.57 ± 0.04	0.69 ± 0.21	0.47 ± 0.22	0.51 ± 0.24
	PET-CAM	0.69 ± 0.04	0.80 ± 0.14	0.59 ± 0.26	0.67 ± 0.08
Lung cancer	U-NET [31]	0.52 ± 0.14	0.64 ± 0.23	0.36 ± 0.19	0.53 ± 0.25
	SegNet [32]	0.60 ± 0.09	0.69 ± 0.14	0.50 ± 0.17	0.57 ± 0.19
	ResUnet [33]	0.59 ± 0.12	0.67 ± 0.17	0.52 ± 0.19	0.57 ± 0.21
	CAMsWPK	0.61 ± 0.07	0.59 ± 0.21	0.57 ± 0.15	0.55 ± 0.24
	MS	0.68 ± 0.17	0.72 ± 0.09	0.54 ± 0.07	0.61 ± 0.03
	CAMs & FCNs	0.59 ± 0.07	0.63 ± 0.12	0.57 ± 0.19	0.57 ± 0.17
	PET-CAM	0.65 ± 0.05	0.65 ± 0.18	0.58 ± 0.15	0.59 ± 0.04

## Data Availability

The dataset used in the study is from the cancer center Henri Becquerel, at Rouen in France. The Institutional Review Board of the Henri Becquerel Center allowed us to access and work on these patients’ data under certain conditions, in accordance with the GDPR framework. If a third party wishes to use the data, please send a specific request to the responsible of the clinical research Unit, Dr. Louis-Ferdinand Pepin, who is the manager of this database, explaining the goal of the study and all persons involved (louis-ferdinand.pepin@chb.unicancer.fr).

## Abbreviations

The following abbreviations are used in this manuscript:

PET	positron emission tomography
MIP	maximum intensity projection
CAD	computer-aided detection
WSL	weakly supervised learning
CNN	convolutional neural network
FCN	convolutional neural networks
NN	Network in Network
CAM	class attention maps
RF	random forests
SVM	support vector machines
SUV	standard uptake value
WPk	without prior knowledge
Ms	manual segmentation
OS	overall survival
ACC	accuracy
Sens	sensitivity
Spec	specificity
AUC	area under the ROC curve
